# A mixed ecologic-cohort comparison of physical activity & weight among young adults from five populations of African origin

**DOI:** 10.1186/1471-2458-14-397

**Published:** 2014-04-24

**Authors:** Amy Luke, Pascal Bovet, Jacob Plange-Rhule, Terrence E Forrester, Estelle V Lambert, Dale A Schoeller, Lara R Dugas, Ramon A Durazo-Arvizu, David A Shoham, Guichan Cao, Soren Brage, Ulf Ekelund, Richard S Cooper

**Affiliations:** 1 Stritch School of Medicine, Loyola University Chicago, Maywood, IL, USA; 2 Institute of Social & Preventive Medicine, Lausanne University Hospital, Ministry of Health, Republic of Seychelles, Lausanne, Switzerland; 3 Kwame Nkrumah University of Science and Technology, Kumasi, Ghana; 4 Tropical Medicine Research Institute, University of the West Indies, Mona, Kingston, Jamaica; 5 Research Unit for Exercise Science and Sports Medicine, University of Cape Town, Cape Town, South Africa; 6 University of Wisconsin, Madison, WI, USA; 7 MRC Epidemiology Unit, Addenbrooke’s Hospital, Cambridge, UK; 8 Department of Sport Medicine, Norwegian School of Sport Sciences, Oslo, Norway

**Keywords:** Physical activity, Obesity, Epidemiologic transition

## Abstract

**Background:**

Examination of patterns and intensity of physical activity (PA) across cultures where obesity prevalence varies widely provides insight into one aspect of the ongoing epidemiologic transition. The primary hypothesis being addressed is whether low levels of PA are associated with excess weight and adiposity.

**Methods:**

We recruited young adults from five countries (500 per country, 2500 total, ages 25–45 years), spanning the range of obesity prevalence. Men and women were recruited from a suburb of Chicago, Illinois, USA; urban Jamaica; rural Ghana; peri-urban South Africa; and the Seychelles. PA was measured using accelerometry and expressed as minutes per day of moderate-to-vigorous activity or sedentary behavior.

**Results:**

Obesity (BMI ≥ 30) prevalence ranged from 1.4% (Ghanaian men) to 63.8% (US women). South African men were the most active, followed by Ghanaian men. Relatively small differences were observed across sites among women; however, women in Ghana accumulated the most activity. Within site-gender sub-groups, the correlation of activity with BMI and other measures of adiposity was inconsistent; the combined correlation across sites was -0.17 for men and -0.11 for women. In the ecological analysis time spent in moderate-to-vigorous activity was inversely associated with BMI (r = -0.71).

**Conclusion:**

These analyses suggest that persons with greater adiposity tend to engage in less PA, although the associations are weak and the direction of causality cannot be inferred because measurements are cross-sectional. Longitudinal data will be required to elucidate direction of association.

## Background

The prevalence of obesity has increased dramatically in most regions of the world over the past three decades, however, the relative importance of the exposures driving this epidemic is still poorly defined [1,2]. Age-related weight gain must result from an imbalance between energy intake and expenditure [3]. The precision of analytic methods to measure either component of the energy balance equation in free-living people has always been problematic, and none of the available methods provide objective estimates of long-term exposure. These methodological limitations may, in part, be circumvented by comparisons among communities where the mean level of obesity varies substantially; in this comparison it must follow that the level of key exposures are also widely different.

Cross-cultural comparisons have played a key role in elucidating the etiology of common chronic diseases [4-9]. For many forms of cancer and cardiovascular disease the causal exposures are widespread within a population and adequate discrimination in life-time exposure between individuals is often beyond the resolving power of epidemiologic methods [10]. Randomized trials are also often not feasible for several reasons. For example, the relevant exposure may be unknown, or participants cannot be ethically randomized to the exposure of interest, or the duration of exposure required may be beyond the realistic time frame for large-scale trials. Many of these classic epidemiologic constraints apply to studies of the etiology of obesity, lending support to cross-cultural comparisons. As is well recognized, however, the ecologic design must control for potential confounding by capturing measurements at the individual level and demonstrating similar relationships between persons as well as between groups. Thus, a mixed ecologic-cohort design is often the most efficient approach to studying this class of conditions.

The research project reported here utilizes a mixed ecologic-cohort design to examine risk factors for obesity involving multiple populations of the African diaspora. Communities across the spectrum of the epidemiologic transition were selected, with substantial background control for genetic factors among the three samples which are of West African ancestry. Using an objective measure of physical activity intensity and duration, i.e., accelerometry, we investigated the relationships between physical activity and adiposity. This report is based on the baseline findings for the five cohorts of young adults that have been recruited for the Modeling the Epidemiologic Transition Study (METS). The sample design was based on community clusters, stratified specifically by prevalence of obesity; our study participants are therefore not representative samples of the countries as a whole.

## Methods

### Sampling design and participant recruitment

Twenty-five hundred adults, ages 25–45, were enrolled in METS between January 2010 and December 2011. A detailed description of the study protocol has been previously published [11]. In brief; five hundred participants, approximately 50% of whom are female, were enrolled in each of five study sites: *viz*, rural Ghana, urban South Africa, the Seychelles, urban Jamaica and metropolitan Chicago. The participants were predominantly of African descent in the five countries. The study sites were selected to represent a broad range of social and economic development as defined by the UN Human Development Index (HDI) 2011: i.e., Ghana as a low middle HDI country [HDI rank 135], South Africa as middle [123], Jamaica [80] and the Seychelles as high [52], and the US as a very high HDI country [4][12]. The samples are not meant to be representative of the countries as a whole but are, however, characteristic of broad lifestyle patterns common to each site.

Exclusion criteria included individuals with infectious diseases, including HIV-positive individuals, and pregnant or lactating women, as well as persons with conditions preventing normal physical activities, e.g. lower extremity disability [11]. In Ghana, a simple random sample was generated for the age-range of the study from the population census for the rural town of Nkwantakese. In Seychelles, sex- and age-stratified random samples were generated from their respective national censuses. In South Africa, the sex- and age-stratified random sample was drawn from previously enumerated areas of Khayelitsha, the third largest township in the country and adjacent to the city of Cape Town. In Kingston, Jamaica, districts were randomly sampled; beginning from a fixed point in each district (e.g., the north-west corner), and door-to-door recruitment was then carried out. Similarly, in Maywood, IL, USA, all city blocks in the community were randomized and door-to-door recruitment was conducted.

The protocol for METS was approved by the Institutional Review Board of Loyola University Chicago, IL, USA; the Committee on Human Research Publication and Ethics of Kwame Nkrumah University of Science and Technology, Kumasi, Ghana; the Health Sciences Faculty Research Ethics Committee of the University of Cape Town, South Africa; the Board for Ethics and Clinical Research of the University of Lausanne, Switzerland; the National Research Ethics Committee of Seychelles; the Ethics Committee of the University of the West Indies, Kingston, Jamaica; and the Health Sciences Institutional Review Board of the University of Wisconsin, Madison, WI, USA. Written informed consent was obtained from all participants.

### Measurements

All measurements were made at outpatient clinics located in the respective communities. Weight (kg) and height (m) measurements were made on all participants while wearing light clothing and no shoes. Weight was measured to the nearest 0.1 kg using the same model standard calibrated balance at all five sites (Seca 770, Hamburg, Germany). Height was measured to the nearest 0.1 cm using a stadiometer (e.g. Invicta Stadiometer, Invicta, London, UK) with the participant’s head held in the Frankfort plane. Waist circumference was measured to the nearest 0.1 cm at the umbilicus and hip at the point of maximum extension of the buttocks. Body mass index (BMI) was calculated as kg/m^2^.

Body composition was estimated by bioelectrical impedance analysis (BIA) with the use of a single-frequency (50 kHz) impedance analyzer (model BIA 101Q; RJL Systems, Clinton Township, MI). A tetrapolar placement of electrodes was used on the right hand and foot. Fat-free mass (FFM) and fat mass (FM) were estimated from measured resistance by using an equation validated in the METS cohorts [13].

Physical activity (PA) was assessed using the Actical accelerometer (Phillips Respironics, Bend, OR, USA). The monitor was worn at the waist, positioned just behind the right hip. Each participant was asked to wear the activity monitor at all times over an 8-day period encompassing the partial first and last days, including during sleep; the only time the monitor should have been removed was while bathing, showering, or swimming. Prior to the initiation of the study, intra-class correlations were calculated to estimate the level of reliability achieved for a given number of days of monitoring in each of our sites. All sites achieved a correlation of at least 0.8 with five days of monitoring, a level of reliability deemed acceptable in many studies [14]. Our protocol allowed for six complete days of monitoring (i.e., after dropping the first and last partial days of wear) thus providing a good level of reliability at 0.83-0.92% for all sites. At present there are no universally applied conventions regarding the definition of sleep-time vs. awake-time for continuously collected, i.e. 24-hours, accelerometry data. For the purpose of the analyses presented here, we assessed activity conducted between the hours of 7 am and 11 pm daily. Raw data downloaded from the accelerometers were first passed through a SAS macro program designed to infer non-wear time from 90 or more minutes of continuous zero activity counts. This criterion was based on visual inspection of the wear/non-wear patterns across a range of different string-length criteria in a subset of files from each country. A valid day of physical activity monitoring was defined as one having 10 or more hours of wear time, i.e. ≥62% of maximal available wear time. Participant files were included for analysis if they contained four or more valid days, i.e. ≥75% of maximum number of days. Sedentary, moderate and vigorous activity levels were defined using published cut-points: sedentary <100 counts per minute (cpm), moderate 1535–3959 cpm and vigorous ≥3960 cpm [15,16]. Using the same protocol employed by the National Center for Health Statistics for the analysis of accelerometry data in the continuous National Health and Nutrition Examination Survey [17], minutes defined as comprising sedentary, moderate, vigorous or moderate plus vigorous activity are presented as the total time in minutes accumulated in either 1- or 10- minute intervals. The 10-minute interval may be considered a modified 10-minute bout as, following prior conventions, we allowed for up to 2 minutes of below threshold count activity before considering the bout to be ended [17]. Data are also presented as activity counts per minute as an overall measure of average physical activity intensity.

### Questionnaires

All questionnaires were administered by centrally trained personnel. Basic health history information, with a focus on cardiovascular conditions and diabetes, was collected, including age of first diagnosis where applicable. Participants were asked about medication and dietary supplement use.

Fifty-four questions were included which covered general household characteristics, participant and significant other’s occupation, parental education and household assets and amenities. These questions were based on the Core Welfare Indicators Questionnaire (CWIQ) from the World Bank, designed to monitor social indicators in low HDI contexts [18]. Employment status was assigned based on whether or not the participant had worked for pay in the previous month, education was coded as a continuous variable and occupations were coded according to standardized categories as manual (technical, service, manual, agriculture or fishing) or non-manual (managerial, professional or clerical) [19,20].

Self-reported physical activity was assessed using the Global Physical Activity Questionnaire (GPAQ; [21]); the GPAQ was developed by the World Health Organization and has been validated for use in developing and industrialized countries. Participants recorded the number of days per week and amount of time per day engaged in moderate and vigorous activities during work, number of days per week and amount of time per day engaged vigorous recreational activities, number of days per week and amount of time per day spent walking or bicycle riding (i.e., travel), and amount of time spent daily in sedentary activities. From these data, average minutes per day spent in activity for work, travel and recreation were calculated.

Data management was centralized at the coordinating center at Loyola University Chicago. All data forms and questionnaires were scanned at each study site and, along with electronic Actical data files, were sent via secure FTP (Bitvise Tunnelier [22]) to the data manager at the coordinating center.

### Statistical analysis

All analyses were conducted in Stata Version 12 (College Station, TX, USA). Statistical significance was accepted at p < 0.05. Descriptive statistics including mean levels and distributions were used to summarize the characteristics of participants in each of the five study sites. Prevalences of health status indicators, i.e. overweight and obesity, were calculated by site for each gender. In addition, descriptive characteristics of the physical activity variables were calculated, including only those individuals with valid data as determined by the inclusion criteria previously described. For normally distributed continuous variables, we used ANOVA and multiple linear regression analyses and chi-squared statistic for categorical variables. Univariate analyses were conducted to determine partial correlation coefficients between parameters of physical activity and adiposity, after adjustment for age. A meta-analysis approach with random effects [23] was undertaken to assess the heterogeneity of the partial regression coefficients across sites for both men and women. A combined estimate was obtained, which is interpreted as the average effect between MVPA and BMI, adjusting for age. For the ecological comparison, mean BMI was regressed on mean MVPA in 1-minute bouts for all sites by gender.

## Results

**Figure 1:**
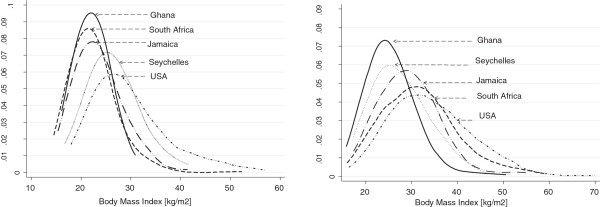
**BMI (kg/m**^**2**^**) distribution by site for men (left panel) and women (right panel).**

The descriptive characteristics of the participants, by site and gender, are presented in Table 1; countries are sequenced in all tables and figures from the lowest HDI ranking (Ghana) to the highest (United States). As a result of the design, mean BMI varied widely across sites, as illustrated by the distributions presented in Figures 1a and b. Men in Ghana and South Africa had similar mean BMIs (e.g. 22 kg/m^2^), although obesity prevalence was slightly higher in South Africa (1.4 vs. 5.5%, respectively; Table 2). Jamaican men were likewise lean (mean BMI = 24). Other measures of adiposity were consistent with the trends in BMI, although the Ghanaian men were 5–6 cm shorter than the other groups, and had lower body weight as well as smaller abdominal circumferences. Among US African-American men, BMI approached 30 kg/m^2^, and almost a third of body weight was estimated to be adipose tissue. Sexual dimorphism in adiposity also varied widely among groups. Ghanaian women, while the leanest of the female cohorts, had a mean BMI of greater than 25, placing more than half them in the overweight or obese categories; South African and Jamaican women had mean BMIs in the range of 30–32, and the mean for US African-American women (i.e., 34) approached the cut-point for Class II obesity (e.g., 35.0-39.9). Among women similar trends in height and waist circumference were observed as among men. Since the mean BMI for five of the site-gender groups falls within the category of “overweight”, differences in prevalence of overweight among groups are blunted, again with the exception of the Ghanaian and South African men. The difference across groups in the prevalence of obesity was stark, ranging from a low of 1% among Ghanaian men to more than 60% for US women.

**Table 1:** Participant characteristics by site and sex – mean ± SD

	**Ghana**	**South Africa**	**Jamaica**	**Seychelles**	**United States**
	**Men**				
Sample size	207	236	249	230	245
Age (y)	34.6 ± 6.7	33.7 ± 5.6	34.0 ± 5.9	36.5 ± 5.1	35.6 ± 6.2
Weight (kg)	63.6 ± 9.1	65.6 ± 13.6	73.1 ± 15.0	80.1 ± 16.0	92.8 ± 24.8
Height (cm)	169.0 ± 6.6	170.9 ± 6.3	176.0 ± 6.7	173.9 ± 6.2	176.6 ± 6.6
Body Mass Index (kg/m^2^)	22.2 ± 2.7	22.4 ± 4.3	23.6 ± 4.5	26.5 ± 4.9	29.7 ± 7.5
Waist Circumference (cm)	77.1 ± 10.5	80.9 ± 11.5	80.3 ± 12.1	89.4 ± 11.8	97.1 ± 21.5
Hip Circumference (cm)	91.8 ± 10.9	94.6 ± 8.4	95.1 ± 9.3	102.8 ± 9.6	109.2 ± 15.9
Fat Mass (kg)	10.6 ± 5.3	15.0 ± 8.3	15.7 ± 9.2	20.6 ± 10.0	29.5 ± 16.6
Body Fat (%)	16.0 ± 6.0	21.7 ± 6.9	20.1 ± 7.4	24.5 ± 7.8	29.7 ± 9.0
Overweight, BMI ≥25 < 30 (%)	13.5	14.8	20.1	37.4	30.6
Obesity, BMI ≥30 (%)	1.4	5.5	9.6	20.9	40.4
Education (y)	9.2 ± 3.8	9.5 ± 2.6	10.6 ± 2.1	13.1 ± 2.4	12.7 ± 1.6
Employed (%)	98.6	91.4	90.8	97.8	85.2
Manual Laborer (%)	60.9	90.4	61.8	54.4	67.1
	**Women**				
Sample size	293	268	251	270	257
Age (y)	34.0 ± 6.6	33.1 ± 6.0	34.7 ± 6.2	35.8 ± 6.0	35.0 ± 6.3
Weight (kg)	63.6 ± 13.1	82.0 ± 22.3	78.5 ± 18.6	72.1 ± 17.3	91.7 ± 24.4
Height (cm)	158.0 ± 5.7	160.1 ± 6.3	163.2 ± 6.6	161.4 ± 6.5	164.0 ± 6.2
Body Mass Index (kg/m^2^)	25.5 ± 5.2	31.9 ± 8.2	29.5 ± 6.7	27.6 ± 6.2	34.1 ± 8.8
Waist Circumference (cm)	84.2 ± 12.5	96.9 ± 16.6	92.0 ± 13.8	87.9 ± 12.4	101.9 ± 19.6
Hip Circumference (cm)	100.2 ± 13.2	114.2 ± 15.6	107.9 ± 11.6	104.4 ± 12.4	117.0 ± 16.0
Fat Mass (kg)	22.8 ± 8.5	36.8 ± 14.8	32.1 ± 12.1	28.1 ± 11.2	42.3 ± 16.4
Body Fat (%)	34.8 ± 6.1	43.4 ± 6.6	39.7 ± 6.1	37.8 ± 6.7	44.6 ± 6.5
Overweight, BMI ≥25 < 30 (%)	32.4	22.4	26.7	30.0	21.4
Obesity, BMI ≥30 (%)	15.7	55.6	45.4	31.1	63.8
Education (y)	7.5 ± 4.1	10.0 ± 2.1	10.7 ± 2.0	13.0 ± ±2.5	13.8 ± 2.6
Employed (%)	89.4	81.7	72.1	95.6	83.7
Manual Laborer (%)	90.4	89.3	68.8	26.4	28.9

**Table 2:** Physical activity parameters as measured using accelerometry during awake time*– mean ± SD

	**Ghana**	**South Africa**	**Jamaica**	**Seychelles**	**United States**
	**Men**				
# Individuals with Valid Data^†^	182	230	223	214	229
Mean Wear Time (hr/d)	15.9 ± 0.6	16.5 ± 0.7	15.5 ± 1.0	14.5 ± 1.3	15.7 ± 0.9
Mean Activity Counts (cpm)	244.8 ± 89.9	253.5 ± 141.4	182.1 ± 93.2	236.5 ± 114.9	200.4 ± 137.6
Sedentary Time (min/d in 1-min bouts)	197.8 ± 43.7	207.3 ± 45.7	224.8 ± 54.7	196.6 ± 52.0	205.5 ± 44.6
Moderate Activity (min/d in 1-min bouts)	44.1 ± 22.8	46.8 ± 27.3	27.0 ± 20.8	31.0 ± 20.1	28.5 ± 28.7
Vigorous Activity (min/d in 1-min bouts)	2.8 ± 3.9	8.7 ± 15.4	2.7 ± 5.5	4.9 ± 7.9	4.5 ± 10.3
MVPA* (min/d in 1-min bouts)	46.9 ± 24.6	55.5 ± 34.6	29.6 ± 23.3	35.9 ± 24.3	33.1 ± 34.7
≥ 30 min/d in 1-min bouts (%)	70.9	76.1	39.5	55.1	38.0
Sedentary Time (min/d in 10-min bouts)	49.7 ± 37.5	48.91 ± 32.7	70.9 ± 48.8	62.5 ± 45.1	48.4 ± 31.2
Moderate Activity (min/d in 10-min bouts)	18.2 ± 14.6	21.3 ± 20.7	9.0 ± 13.3	10.4 ± 11.8	13.8 ± 22.1
Vigorous Activity (min/d in 10-min bouts)	1.0 ± 2.3	4.8 ± 13.8	1.1 ± 3.9	2.1 ± 5.6	2.5 ± 8.5
MVPA (min/d in 10-min bouts)	21.9 ± 16.7	31.6 ± 28.7	12.4 ± 16.3	16.4 ± 16.8	19.5 ± 29.9
≥ 30 min/d in 10-min bouts (%)	30.2	42.6	11.7	19.6	20.5
	**Women**				
# Individuals with Valid Data	269	264	234	238	242
Mean Wear Time (hr/d)	15.9 ± 0.7	16.6 ± 0.6	15.6 ± 0.9	14.4 ± 1.4	15.6 ± 1.0
Mean Activity Counts (cpm)	183.0 ± 66.8	138.3 ± 61.5	143.1 ± 61.5	170.3 ± 70.5	136.2 ± 76.0
Sedentary Time (min/d in 1-min bouts)	193.3 ± 38.4	220.2 ± 44.1	208.4 ± 43.5	185.7 ± 48.1	205.3 ± 46.8
Moderate Activity (min/d in 1-min bouts)	25.5 ± 16.3	21.1 ± 15.1	19.0 ± 14.7	22.0 ± 13.9	13.9 ± 16.1
Vigorous Activity (min/d in 1-min bouts)	0.4 ± 0.9	0.9 ± 2.4	0.5 ± 2.0	0.9 ± 2.1	1.3 ± 3.2
MVPA* (min/d in 1-min bouts)	25.9 ± 16.7	22.0 ± 16.3	19.5 ± 15.3	22.9 ± 14.7	15.1 ± 17.7
≥ 30 min/d in 1-min bouts (%)	35.3	23.9	23.1	27.3	14.5
Sedentary Time (min/d in 10-min bouts)	43.4 ± 26.2	58.3 ± 36.2	53.2 ± 32.6	50.6 ± 35.9	46.3 ± 31.3
Moderate Activity (min/d in 10-min bouts)	10.1 ± 10.3	9.3 ± 10.0	7.1 ± 8.0	9.2 ± 9.2	5.0 ± 11.5
Vigorous Activity (min/d in 10-min bouts)	0.1 ± 0.5	0.3 ± 1.4	0.2 ± 1.5	0.2 ± 1.2	0.7 ± 2.5
MVPA (min/d in 10-min bouts)	10.5 ± 10.6	10.2 ± 11.3	7.7 ± 8.9	10.3 ± 10.2	6.6 ± 13.3
≥ 30 min/d in 10-min bouts (%)	7.1	5.7	2.1	5.9	5.4

*Moderate plus vigorous activity.

^†^# Individuals with Valid Data: At least 4 days with 10+ hours of wear.

**Figure 2:**
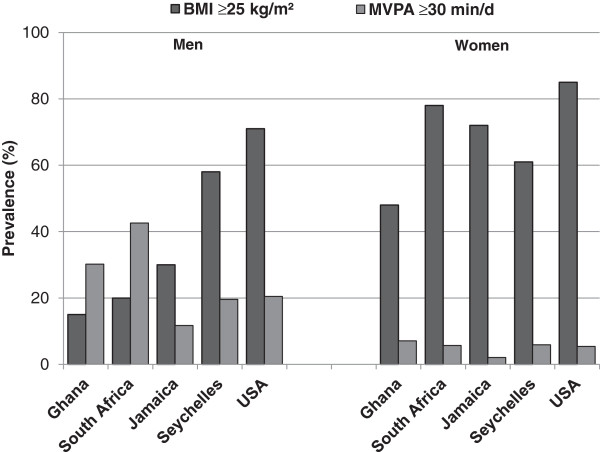
Cumulative prevalence of overweight plus obesity (BMI ≥ 25) and proportion of participants meeting physical activity recommendations (moderate-to-vigorous activity in bouts of at least 10 minutes ≥30 min/d) by site and gender.

The prevalences of BMI ≥25, i.e., overweight plus obesity, are presented graphically in Figure 2, along with the proportion of participants who met physical activity guidelines [24] of at least 30 minutes per day of MVPA recorded in bouts of at least 10 minutes. Far fewer women than men met recommendations for MVPA.

Activity levels as measured by accelerometry were summarized by current standard methods [17], including average counts for the entire period of recording and continuous minutes spent in 1- and 10-minutes bouts of moderate-to-vigorous activity (MVPA); sedentary time was also summarized in 1- and 10-minute bouts (Table 2). Overall, 93% of the participants provided complete accelerometry data as defined by having valid wear time for at least 10 hours per day on a minimum of 4 days of the measurement period; the proportion of participants with complete data ranged from 86% among women in the Seychelles to 99% among South African women. The South African men had the highest mean activity levels (cpm), followed by Ghanaians, and they also spent the largest proportion of the day in 10-minute bouts of MPVA (i.e., 32 minutes among South Africans vs. 12 among Jamaicans). Women in the communities from Ghana and the Seychelles had the highest levels of activity levels (183 and 170 cpm, respectively), with considerably lower levels recorded among the other three groups (e.g., all less than 145 cpm). Accumulated time spent in MVPA was very low for all women; there was essentially only one 10-minute bout per day for Ghanaian, South African or Seychellois women while neither the Jamaican nor the US women registered even a single 10-minute bout. Because the absolute levels were so low, few differences are relevant; however, it is worth noting that both the accumulated time in MVPA - time spent in 1-minute and 10-minute bouts of MVPA - were lowest in women from Chicago. The proportion of participants who accumulated at least 30 minutes of MVPA per day in 1-minute bouts averaged 55.9% among men and 24.8% among women. It is, however, recommended that to be of physiologic benefitl MVPA should be obtained in bouts lasting at least 10 minutes [25]. Using this metric, only 25% of men and 5% of women met the recommendations for MVPA.

**Figure 3:**
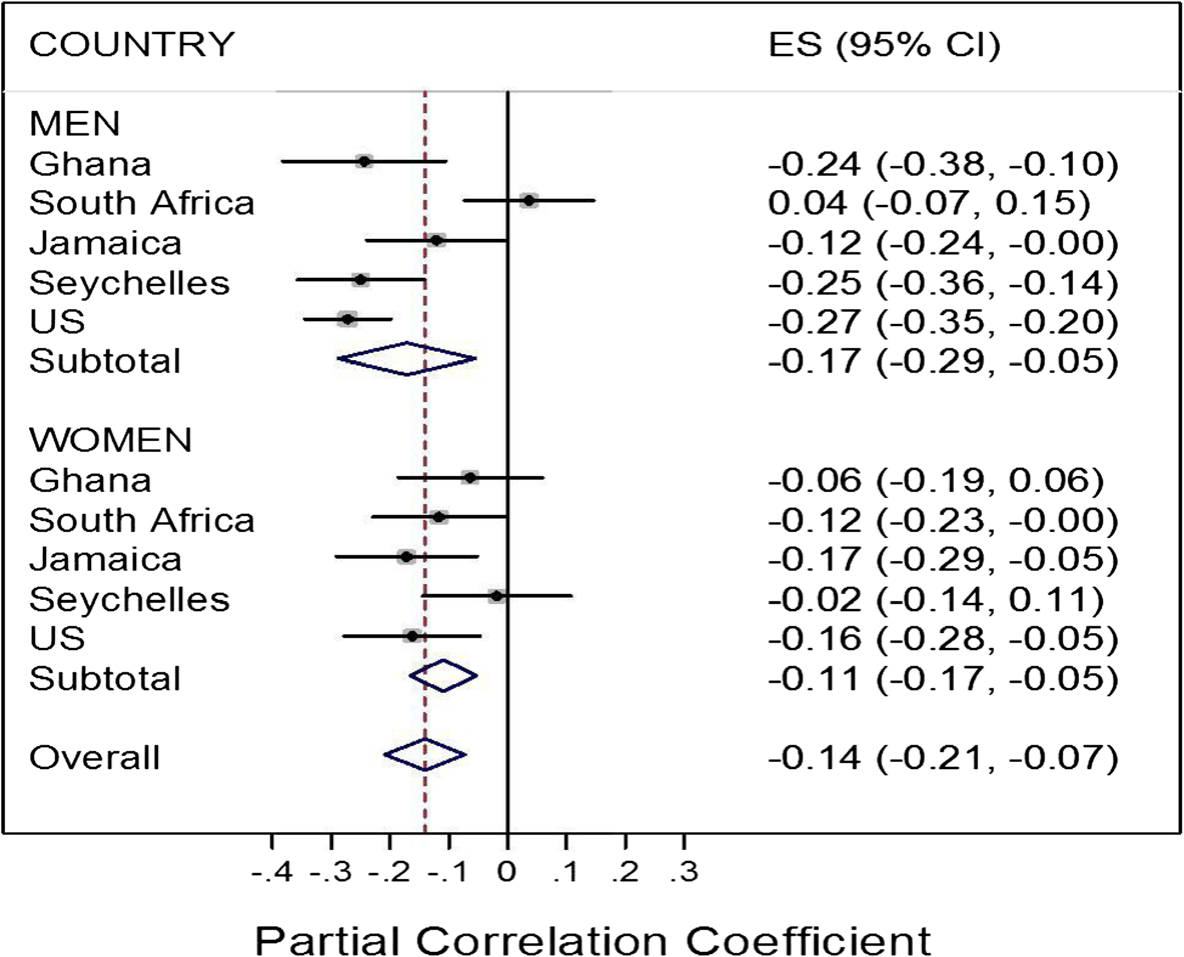
Forest plots describing partial correlation coefficients for relationship between MVPA and BMI among participants enrolled in METS: men overall r = -0.17, women overall r = -0.11, combined overall r = -0.14.

Correlations were assessed between average intensity (cpm) in 1-minute and 10-minute bouts of MVPA (min/d) with BMI, waist circumference and percent body fat (Table 3). Among men, with the exception of the samples from Cape Town and Kingston, statistically significant negative associations were observed for each of the adiposity measures. Among women, although usually negative, the correlations were much less consistent and, in general, not significant. Sedentary time was not positively correlated – as might be anticipated - with any adiposity-related parameter in men or women. Figure 3 illustrates the overall partial correlation between BMI and one activity measure, ie, MVPA in 1-minute bouts, for men and women, respectively. There was significant heterogeneity between sites for men (p <0.001), but not for women (p-value = 0.35). The estimated combined partial regression coefficient was -0.17 (95%CI = -0.29,-0.06), and -0.11 (95%CI = -0.17, -0.05) for men and women, respectively. And while there are dangers for exaggerated associations when combining heterogeneous subsets, the partial regression coefficient for men and women combined was intermediate between those of men and women (-0.14, 95%Cl -0.21, -0.07).

**Table 3:** Partial correlation coefficients between physical activity parameters & measures of adiposity by site & sex

	**Ghana**			**South Africa**			**Jamaica**			**Seychelles**			**United States**		
	**BMI**	**Waist**	**% Fat**	**BMI**	**Waist**	**% Fat**	**BMI**	**Waist**	**% Fat**	**BMI**	**Waist**	**% Fat**	**BMI**	**Waist**	**% Fat**
**Men**															
Sample Size	180			230			223			214			229		
Mean Activity Counts (ct/min)	-0.28***	-0.21**	-0.36***	0.04	-0.01	0.02	-0.11	-0.14*	-0.17*	-0.22***	-0.26***	-0.26***	-0.24***	-0.23***	-0.30***
MVPA** (min/d in 1-min bouts)	-0.25***	-0.17*	-0.30***	0.04	0.03	0.04	-0.12	-0.12	-0.16*	-0.24***	-0.28***	-0.27***	-0.27***	-0.24***	-0.33***
MVPA (min/d in 10-min bouts)	-0.17*	-0.15*	-0.21**	0.08	0.08	0.11	-0.11	-0.11	-0.13	-0.27***	-0.30***	-0.30***	-0.25***	-0.24***	-0.30***
Sedentary Time (min/d in 1-min bouts)	0.11	0.06	0.14	0.08	0.03	0.05	0.02	0.03	0.04	0.02	-0.08	-0.06	-0.03	-0.06	-0.06
**Women**															
Sample Size	269			264			232			237			241		
Mean Activity Counts (ct/min)	-0.14*	-0.18**	-0.19**	-0.08	-0.05	-0.07	-0.17*	-0.17**	-0.15*	0.003	0.01	-0.03	-0.12	-0.11	-0.14*
MVPA** (min/d in 1-min bouts)	-0.06	-0.13*	-0.11	-0.12*	-0.08	-0.09	-0.15*	-0.16*	-0.13	-0.01	-0.02	-0.04	-0.16*	-0.14*	-0.20**
MVPA (min/d in 10-min bouts)	-0.08	-0.10	-0.12*	-0.10	-0.05	-0.07	-0.09	-0.09	-0.07	-0.01	-0.04	-0.01	-0.11	-0.10	-0.15*
Sedentary Time (min/d in 1-min bouts)	0.005	-0.03	-0.04	-0.07	-0.13*	-0.15*	-0.07	-0.03	-0.06	-0.01	-0.07	-0.09	0.02	0.01	-0.07

*Significant at p < 0.05.

**Significant at p < 0.005.

***Significant at p < 0.001.

Ecological associations were examined based on mean values of BMI and minutes per day of MVPA in 1-minute bouts. Based on these aggregate measures, the negative correlation was -0.91 for women and -0.61 for men. The magnitude of the ecological associations between BMI and other parameters of physical activity, i.e., average intensity (cpm) or MVPA in 10-minute bouts, were similar with women consistently exhibiting stronger correlations than men between sites although, as previously noted, within-site associations were much weaker. All associations were similar when using the 60-minute zero string criteria for detection of accelerometer non-wear (data not shown).

Results from the GPAQ are presented in Table 4. Congruent with objectively measured MVPA, Ghanaian men and women reported the highest levels of work-related activity. In contrast, Seychellois participants reported the lowest levels of activity across all 3 principal domains of the GPAQ - occupation, travel and recreational.

**Table 4:** Self-reported physical activity associated with work, travel and recreation and sedentary behavior as assessed by the global physical activity questionnaire (median [range] minutes/day)

	**Ghana**	**South Africa**	**Jamaica**	**Seychelles**	**United States**
	**Men**				
Work-related Moderate-Vigorous Activity	330 (6–925)	120 (2–720)	137 (1–823)	51 (1–720)	171 (2–1029)
Travel	51 (3–600)	51 (4–480)	45 (1–514)	21 (2–171)	31 (1–780)
Recreation Vigorous Activity	25 (1–214)	86 (3–420)	34 (2–240)	26 (4–129)	39 (1–600)
Sedentary Behavior	120 (0–840)	420 (0–900)	300 (15–1080)	300 (60–900)	360 (60–1080)
	**Women**				
Work-related Moderate-Vigorous Activity	180 (6–780)	111 (4–780)	60 (1–1029)	43 (2–617)	56 (1–823)
Travel	43 (3–617)	43 (2–600)	30 (1–600)	21 (3–180)	21 (1–480)
Recreation Vigorous Activity	13 (1–240)	64 (9–540)	28 (4–720)	17 (3–86)	26 (2–335)
Sedentary Behavior	150 (0–1200)	510 (30–860)	300 (20–1020)	300 (30–780)	420 (30–1140)

## Discussion

The international comparisons described here demonstrate site-specific negative associations at both the individual and group level between activity captured by accelerometry and measures adiposity. While the individual-level associations are small in magnitude, there is moderate consistency across groups with statistically significant associations being found among men, with the exception of South Africa, among the women, however, associations did not achieve significance. Quantitatively these associations were small in magnitude, as one might expect for measurements of a fairly short time sample for habitual physical activity. It should be noted, however, that four days of activity monitoring has been shown to be sufficient to capture typical patterns among adults [14]. The strengths of the observed associations are similar to those previously reported using data nationally representative for the United States [26,27].

In general ecological associations should be interpreted as a permissive construct, i.e., if they are absent then the hypothesis being tested is unlikely to be true. In this study, therefore, group level associations support the hypothesis of a pathway from activity to obesity: mean physical activity was lowest in the groups with the highest mean BMIs.

While ecological comparisons may provide a quantitative view of associations between adiposity and measures of physical activity in the broadest sense, it is the non-quantifiable factors that provide the context for those associations. According to self-report, there were relatively minor differences between men or women across the five sites with regard to amount of time spent in travel or in recreational activities. There were, however, stark differences in reported work-related MVPA, with both men and women in Ghana reporting much higher median levels than any of the other sites. For women in METS, there is an almost two-fold difference in measured MVPA and three-fold differences in self-reported MVPA between the least active and the most active cohorts and part of this difference is likely due to types of primary employment within each site. The very low MVPA levels and, possibly, the very high mean BMI observed among the US women may well be related to the fact that they report the lowest levels of manual labor with most of their employment taking place in the healthcare, service representative and education fields (76% of the cohort are engaged in these occupations). In contrast, among Ghanaian women who have both the highest measured MVPA and lowest mean BMI, 90% report being involved in manual labor, primarily trading (40%) and agriculture (21%). Among the other three cohorts of women, the measured MVPA differed very little and the levels of adiposity also were not dramatically different. In both South Africa and Jamaica, the women were overwhelmingly engaged in routine service occupations and trading (>50% in each site), while among the Seychellois, there was a much higher proportion of clerical and professional occupations reported (~48%).

The straightforward explanation that differences in activity and adiposity may be primarily due to differences in occupation, however, does not hold true for the men. Jamaican men have the lowest levels of MVPA, the lowest overall physical activity as measured by counts per minute and the second lowest work-related self-reported MVPA, and yet they are very lean. Moreover, among Jamaican men at least one-third of the cohort was reportedly engaged in the construction industry. In contrast, men in the US have higher objectively measured MVPA, higher work-related MVPA, with a comparable proportion in manual labor occupations, yet are much more obese. Among the male cohorts, there are instances where the data are consistent across domains, i.e., Ghanaian men are lean and have relatively high levels of both objectively measured MVPA and self-reported MVPA; about one-quarter of the cohort works as subsistence farmers. The interplay between physical activity, adiposity, occupation and environment is clearly complex and undoubtedly influenced by other unexplored contextual factors.

Although associations between physical activity and BMI were observed in this study as well as in others [28,29], the design prevents any assumption of causality. Other studies conducted over the last 5 to 10 years provide a body of data which suggests that reduction in physical activity has a minor or non-existent role in the etiology of the contemporary obesity epidemic [1,2,27,30,31]. Moreover, reverse causality must also be postulated. Persons with greater body mass may have consequently reduced walking and other activities requiring use of large muscle groups, which is the principal domain captured by accelerometry [32,33]. Examples of weak to non-existent associations include our previous study of a smaller sample of women from southwest Nigeria and metropolitan Chicago – with measurement of physical activity-related energy expenditure using doubly labeled water; we did not find either individual-level or group-level negative associations between energy expended in activity and BMI or adiposity [27,34,35]. A recent meta-analysis of data from cultures with low levels of mechanization likewise recorded similar amounts of energy expended as observed in industrialized societies [30]. Likewise, recent studies of the Hadza people of Tanzania, who subsist by foraging and hunting [31], and the Tsimane, forager-horticulturalists of Amazonian Bolivia [36], reported little difference between energy expenditure or physical activity levels by degree of modernization or income. Compared to women in high-income populations, Hadza women had no excess expenditure in daily activity, as assessed by doubly labeled water, yet had lower BMIs [31]. Physical activity levels determined through direct observation and accelerometry in a subsample among the Tsimane showed little variation regardless of degree of “modernization” [36]. Additionally, levels of physical activity were found to be comparable between Europeans and urban Cameroonians when measured using individually calibrated, combined heart rate and motion sensors [37-39], although rural Cameroonians and rural Kenyans recorded higher activity than their urban counterparts [37].

Although the technology and design sophistication for studies of energy expenditure in free-living individuals has been improving rapidly, to our knowledge, all studies reported to date either included small sample sizes (e.g., N = 19 among the Hadza [31] and N = 16 urban Cameroonians [37], or comparisons based on independent studies which did not share a standardized protocol [30]. It also must be appreciated that the different methodologies employed, e.g., doubly labeled water, accelerometry, heart rate monitoring, direct observation or others, measure very different domains of human metabolism and activity; although correlated, physical activity energy expenditure is a different component of physical activity than body movement measured by accelerometry. In addition, the imbalance in energy intake and expenditure can be small in the short run, but result in large life-time excess weight gain, thereby challenging the precision of our measurement tools [40-42].

The need for objective measurement of physical activity in the effort to understand the relationship between movement or physical activity energy expenditure and excess weight gain is obvious when one considers that most previous data were obtained using questionnaires and surveys, which have very limited precision [43]. According to the World Health Organization’s STEPwise Approach to Chronic Disease Risk Factor Surveillance Program, over 83% of men and 75% of women in 22 African countries met the organization’s physical activity recommendations [44]. Although the countries included and the metrics used are not identical, overall in our five cohorts only 56% of the men and 25% of the women recorded 30 minutes/day or more of MVPA in 1-minute bouts on at least 4 days of the measurement period; a much smaller percentage recorded the 30 minutes/day of MVPA in 10-minute bouts (39% and 14% for men and women, respectively). The countries surveyed as part of the STEPwise program may not be directly compared to ours because only the Seychelles was included in both studies. In the Seychelles, however, we found only 12% of our cohort met the recommended ≥30 minutes/day of MVPA in 10-minute bouts where as the STEPwise figure was >81% [44]. Using the WHO’s survey data to understand the association between activity and obesity would not be useful, at least among African populations.

## Conclusions

In summary, we have demonstrated very modest negative correlations between adiposity and movement in five heterogeneous cultural settings, associations which exist at both the individual and group levels. Crucial caveats, however, apply in the interpretation of these findings. First, as clearly noted, the cross-sectional nature of the data limits our ability to make causal inferences and determine the direction of association. It could be that the excess weight limits time spent in physical activity due to increased workloads is just as possible as the decreased levels of physical activity are causing excessive weight gain. The METS protocol involves a longitudinal component so an anticipated follow-up period of up to 5 years will address this shortcoming. Second, accelerometry measures body movement primarily in the vertical direction, most pronounced while walking, standing up and similar whole-body activities; the correlation with energy expenditure in activity measured by doubly labeled water is approximately 0.5 [45]. Low precision of the measurement of exposure variables, however, should not create consistent negative associations, but rather bias the findings toward the null. Third, the data presented here in no way preclude the possibility that excess intake, relative to expenditure, is also occurring in some or all of these communities, and may be making a larger contribution to excess adiposity. Given the current state of knowledge, it is reasonable to leave open the hypothesis that in some settings and in some stages of cultural evolution, decreased energy expenditure may play a role, while in other settings and stages it may not**.** What is crucial, however, is that public health recommendations should be formulated on the basis of clear-cut empirical evidence so that they will have maximum impact on policy interventions to prevent obesity in future generations and, if possible, aid in the process of weight loss among those who are currently obese. Acquisition of an adequately comprehensive data base to underpin these recommendations is therefore an urgent challenge in epidemiology.

## Competing interests

The authors declare that they have no competing interests.

Financial competing interests.

The authors have no financial competing interests to declare. METS is funded in part by the National Institutes of Health (1R01DK80763).

Non-Financial competing interests.

The authors have no non-financial competing interests to declare.

## Authors’ contributions

AL, TEF, EVL, JPR, PB, RDA, RSC all conceived the idea and contributed equally to the manuscript. RADA, LRD, DAS, SB, UE and GC all performed the analysis, tables and figures. All authors (AL, TF, EVL, JPR, PB, RADA, LRD, DAS, GC, DS, SB, UE & RSC) contributed equally to the interpretation of the data and writing of the manuscript.

## Pre-publication history

The pre-publication history for this paper can be accessed here:

http://www.biomedcentral.com/1471-2458/14/397/prepub
